# Postoperative Mechanical Bowel Obstruction Secondary to Jackson-Pratt (JP) Drain Placement in a Patient With Perforated Appendicitis

**DOI:** 10.7759/cureus.91670

**Published:** 2025-09-05

**Authors:** Yatin Srinivash Ramesh Babu, Krina J Patel, Jesse Obregon, Olaf Kahane, Gerardo Kahane, Orlando Morejon

**Affiliations:** 1 Medicine, Dr. Kiran C. Patel College of Osteopathic Medicine, Nova Southeastern University, Fort Lauderdale, USA; 2 Medicine, Dr. Kiran C. Patel College of Allopathic Medicine, Nova Southeastern University, Fort Lauderdale, USA; 3 General Surgery, HCA Florida Kendall Hospital, Miami, USA; 4 College of Medicine, Florida State University, Tallahassee, USA; 5 General Surgery, Aventura Hospital and Medical Center, Aventura, USA; 6 General Surgery, Kendall Regional Medical Center, Miami, USA

**Keywords:** general surgery, jp drain, mechanical bowel obstruction, perforated appendicitis, surgical case report

## Abstract

Postoperative mechanical bowel obstruction is an infrequent but significant complication following abdominal surgeries. The use of Jackson-Pratt (JP) drains (Cardinal Health, 7000 Cardinal Place, Dublin, Ohio, 43017), while effective in the prevention of fluid collections, may contribute to intestinal obstruction in rare cases. We present the case of a 50-year-old female patient with perforated appendicitis who underwent a robotic appendectomy with intraoperative JP drain placement. Her recovery gradually became complicated by progressive abdominal distention, bilious emesis, and elevated nasogastric tube output. Despite initially unremarkable postoperative radiographs, CT imaging on postoperative day 5 revealed a small bowel obstruction with a transition point near the JP drain. Exploratory laparotomy confirmed bowel entrapment around the drain, with resolution upon drain removal and adhesion lysis, and subsequent full recovery postoperatively.

This case highlights the potential for JP drains to induce mechanical bowel obstruction, underscoring the importance of careful drain placement, postoperative monitoring for obstructive symptoms, and maintaining a high index of suspicion for drain-related complications in patients with delayed postoperative recovery. As clinically appropriate, alternative drain management strategies should be considered for reducing the risk of obstruction.

## Introduction

Perforated appendicitis is a severe sequelae of acute appendicitis, occurring in approximately 13-20% of cases [1]. It is denoted as a breach in the layers of the appendiceal wall, which subsequently results in the emptying of intestinal contents into the peritoneal cavity [2]. The current standard of care in practice is urgent surgical intervention, often accompanied by the placement of intra-abdominal drains to mitigate postoperative fluid collections and the development of possible abscesses [3].

Jackson-Pratt (JP) drains (Cardinal Health, 7000 Cardinal Place, Dublin, Ohio, 43017) are widely applied in abdominal operations, particularly in perforated appendicitis, to drain fluid and monitor for possible complications [4]. JP drains are favored for their closed suction mechanism, relying on gravity or negative pressure to facilitate drainage and ultimately minimizing retrograde infection [5]. Although relatively safe, JP drains can occasionally lead to rare yet severe complications, including mechanical bowel obstruction, among others such as foreign body reaction, delayed wound closure, and increased risk of fistula formation [6].

Mechanical obstruction in the postoperative period caused by JP drains is a complication with an incidence of less than 1% [7]. Reasons for the obstruction can include adhesion of the bowel to the drain, entrapment of bowel loops in the side holes of the drain, and compression of intestinal loops by the drain [8]. The clinical presentation typically occurs with pain in the abdomen, distension, nausea, vomiting, and obstipation, typically in the first week after surgery [9].

The differential diagnoses of post-surgical bowel obstruction include adhesive small bowel obstruction, internal herniation, anastomotic stricture, and postoperative ileus [10]. Additionally, associated factors such as malnutrition, prior abdominal surgeries, and inflammatory bowel disease may also predispose patients towards an increased risk of drain-related complications [11].

This case report is intended to highlight the rare manifestation of JP drain-induced mechanical obstruction as a consequence of emergency surgery for perforated appendicitis, stressing the significance of keeping a high index of suspicion for this issue in any patients with symptoms of postoperative obstruction.

## Case presentation

A 50-year-old woman with no reported past medical or surgical history presented to our emergency department complaining of three days of right lower quadrant abdominal pain with associated nausea, vomiting, and diarrhea. On arrival, the patient was hemodynamically stable and afebrile. Physical exam revealed tenderness to palpation of the right lower quadrant at McBurney’s point with no guarding, rebound tenderness, or rigidity noted. Laboratory results were significant for leukocytosis of 12,000 cells/µL (reference range: 4,000cells/µL- 10,500 cells/µL).

**Figure 1:**
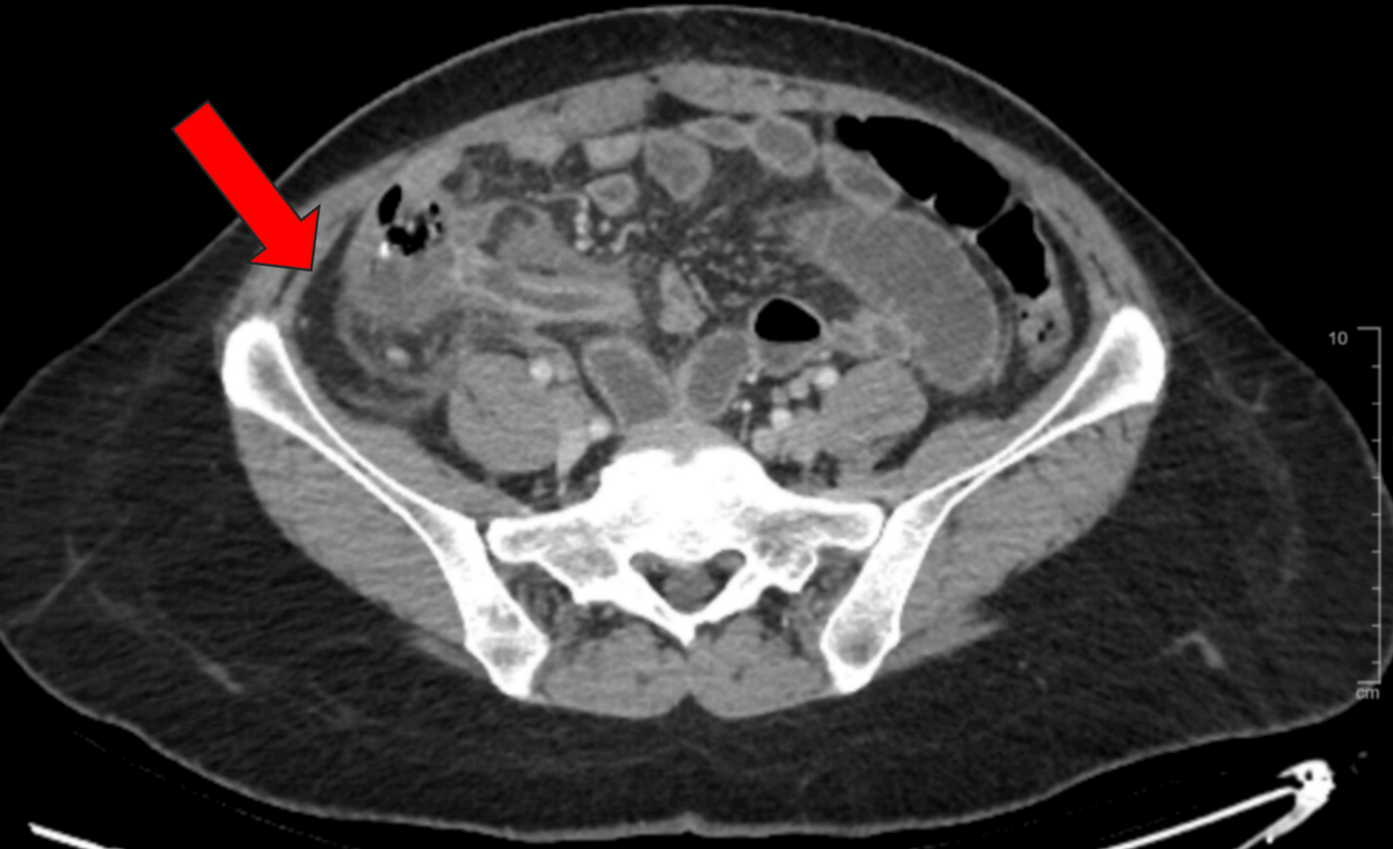
Computerized tomography (CT) imaging of the abdomen and pelvis with IV contrast demonstrating a dilated appendix measuring 1.2 cm with diffuse mucosal thickening and surrounding inflammatory changes. Arrow size: 2.5 cm x 0.6 cm

Computerized tomography (CT) imaging of the abdomen and pelvis with IV contrast demonstrated a dilated appendix measuring 1.2 cm with diffuse mucosal thickening and surrounding inflammatory changes, as shown in Figure 1. However, there was no evidence of perforation, abscess formation, free air, or free fluid. Diffuse small bowel dilation without an abrupt transition point was also noted on imaging, concerning for a reactive ileus. After a discussion with the patient, she consented to a robotic appendectomy, scheduled for the next morning. The patient was initiated on piperacillin and tazobactam for preoperative antibiotic coverage and prepped for surgery.

The following morning, we proceeded with a robotic appendectomy. Pneumoperitoneum was established with a Veress needle in the left upper quadrant to 15 mmHg. Entrance into the abdomen was accomplished under direct visualization using an optiview technique with an 8 mm robotic port in the left upper quadrant. An 8 mm robotic port was placed in the epigastric region along with an 8 mm port in the left lower quadrant, after which the robot was docked and appendectomy was begun. At the time of surgery, the right lower quadrant was noted to be heavily inflamed with purulent peritonitis. The appendix was visualized and noted to be adhered to a nearby loop of small bowel, with a small perforation visible at the center of the appendix. The appendix was grasped and separated from the adhered small bowel with gentle blunt dissection. The mesoappendix was taken using a vessel sealer all the way to the base of the appendix and stapled across the base using a 45 mm blue load stapler. The abdomen was then irrigated and suctioned to remove any residual free fluid. Upon removal of the appendix from the abdomen, we placed a 15 French JP drain (Cardinal Health, 7000 Cardinal Place, Dublin, Ohio 43017) in the right lower quadrant, which was brought up through the skin from the left lower quadrant trocar site. The remainder of the skin incisions were closed, and the patient was uneventfully extubated and transferred to the Post Anesthesia Care Unit in stable condition. 

On postoperative day one, the patient was started on a regular diet and reported feeling well. Vitals were stable, laboratory findings were unremarkable, and the Blake drain was noted to have 180 cc of serosanguineous drainage since surgery. The patient remained hospitalized for continued intravenous antibiotic therapy. 

On postoperative day two, the patient had several episodes of bilious emesis overnight and complained of abdominal pain. On physical exam, her abdomen appeared distended but without signs of peritonitis. Given these events, a nasogastric (NG) tube was placed that morning.

Over the next two days, the patient reported improvement in symptoms but was noted to have continued high bilious output from her NG tube, totaling greater than 1400 cc in 24 hours on both days. She reported passing flatus but denied any bowel movements since before surgery. During this time, the JP drain was functioning appropriately, averaging approximately 150 cc of serosanguinous output daily. 

**Figure 2:**
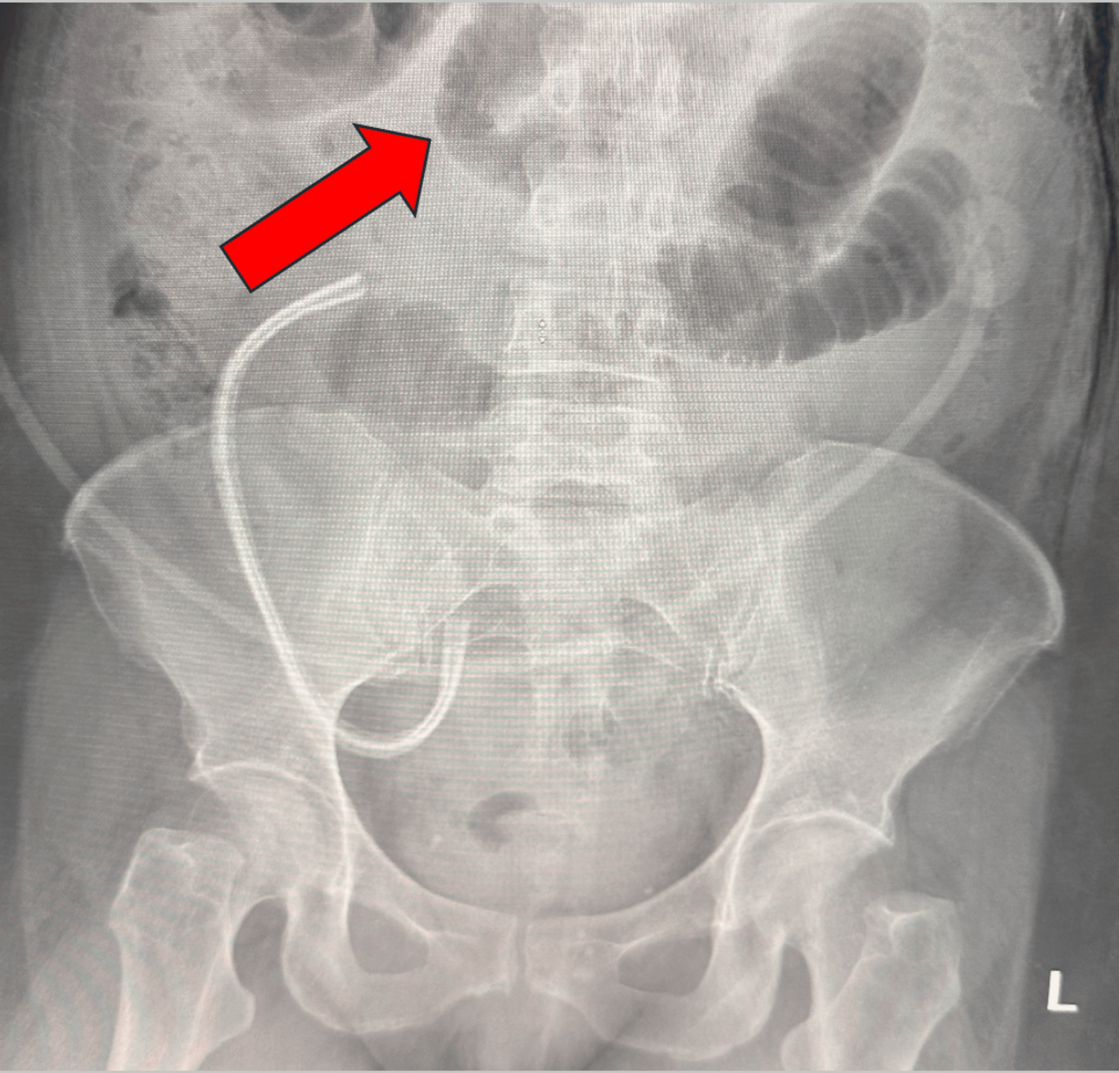
KUB radiograph showing diffuse small bowel dilation consistent with a postoperative ileus. Arrow size: 3.2 cm x 0.8 cm KUB: Kidney, urinary, bladder

As part of her work-up to identify an etiology for the high NG tube output, a kidney, urinary, bladder (KUB) radiograph was ordered on postoperative day three, which showed diffuse small bowel dilation consistent with a postoperative ileus, as shown in Figure 2. Conservative treatment was continued for the next two days with persistently high output from her NG tube and no bowel movement. 

**Figure 3:**
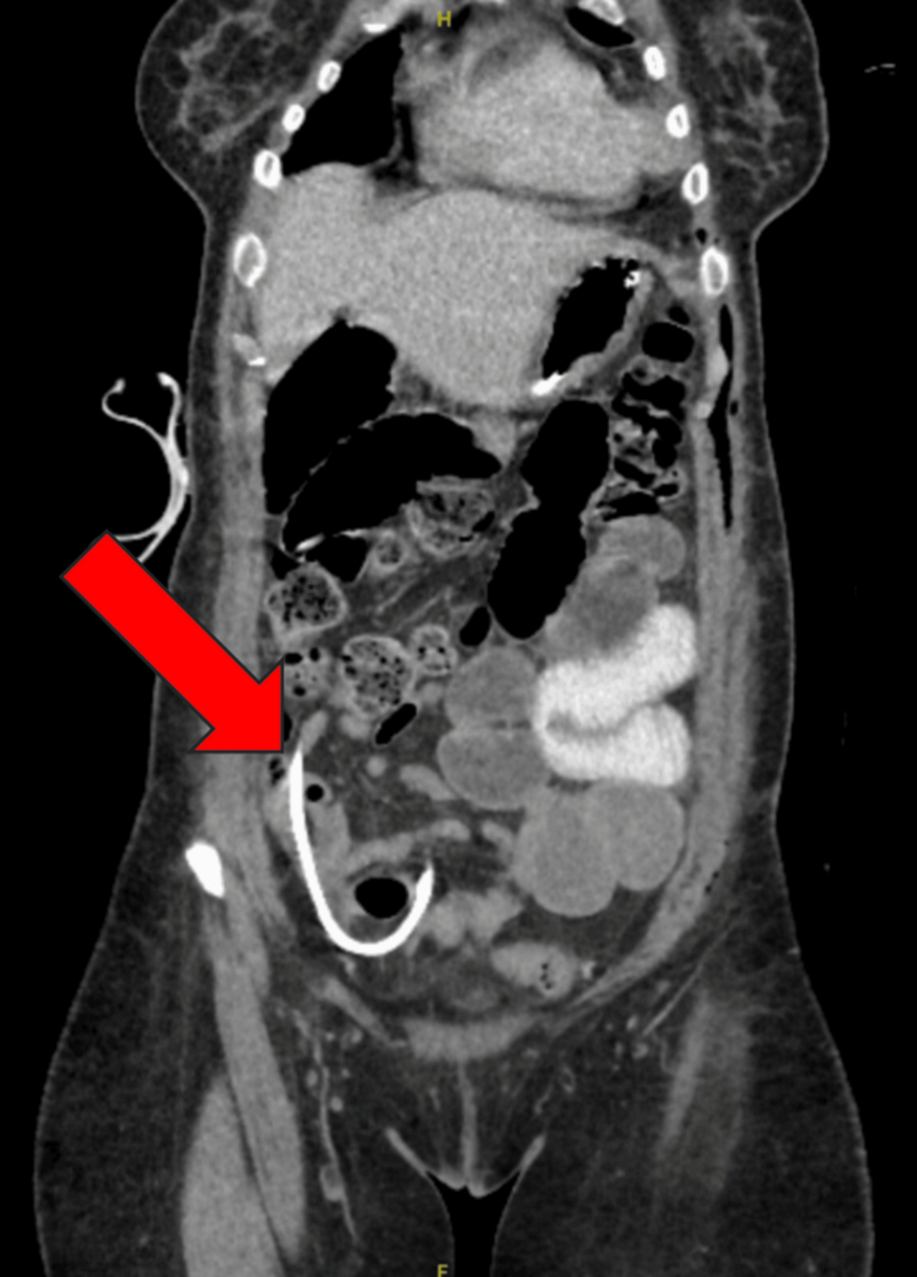
CT abdomen and pelvis with PO contrast, revealing small bowel dilation with a transition point in the right lower quadrant with associated mesenteric swirling. PO: per os (oral) Arrow size: 3.2 cm x 0.8 cm

On postoperative day five, a CT abdomen and pelvis with PO contrast was performed, revealing small bowel dilation with a transition point in the right lower quadrant with associated mesenteric swirling, as shown in Figure 3. Given these findings and lack of improvement in the clinical course, the patient underwent an exploratory laparotomy. 

**Figure 4:**
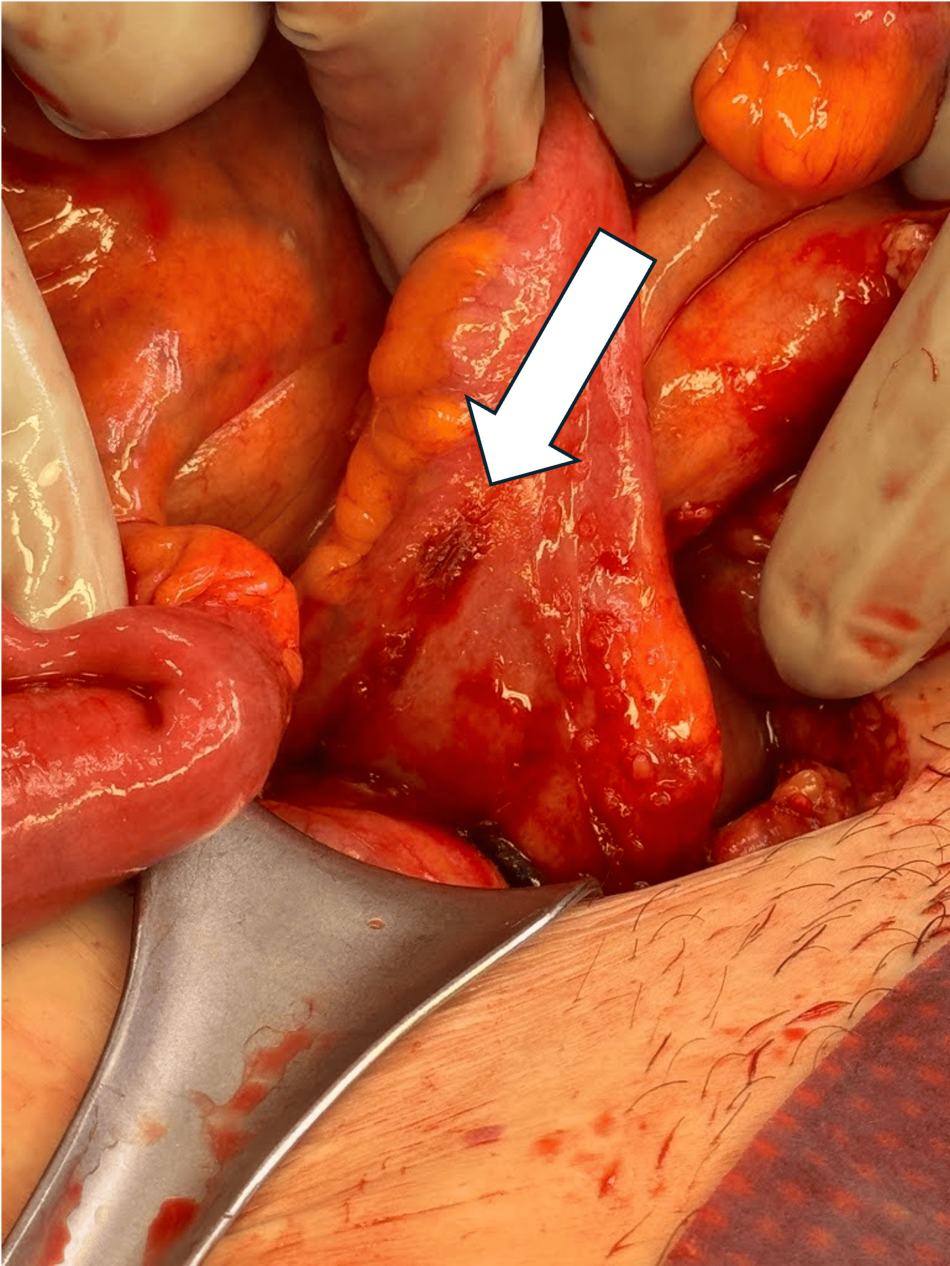
Visible imprint of JP drain on small bowel, as shown by the arrow. Drain serves as a “maypole” around which the small bowel twists. Arrow size: 3.0 cm x 0.75 cm JP: Jackson-Pratt

For the exploratory laparotomy, a midline infraumbilical skin incision was used to enter the abdomen, and we proceeded to eviscerate the small bowel. The proximal small bowel was dilated, and as we worked more distally, we noted a loop of distal small bowel to be adhered and wrapped around our previously placed JP drain. The JP drain was removed, and the adhesions were sharply lysed, relieving the mechanical obstruction. The affected bowel was inspected and noted to be healthy and well-perfused. However, an imprint of the JP drain was visible on the bowel, as shown in Figure 4.

As inspection of the remainder of the small bowel was unremarkable, the abdomen was subsequently irrigated and closed. An NG tube was left in place, and the patient was extubated uneventfully.

The patient’s second postoperative course was uneventful. Following the return of bowel function, her NG tube was discontinued on postoperative day two. By postoperative day four, the patient was tolerating a regular diet and was discharged home. A clinic follow-up with the patient occurred two weeks later, and she reported feeling well with no complaints. 

## Discussion

Mechanical small bowel obstruction due to JP drain entrapment is an infrequent but significant postoperative complication. This case underscores a rare instance in which a JP drain contributed to small bowel obstruction following a robotic appendectomy for perforated appendicitis. While the placement of JP drains in the management of intra-abdominal collections and prevention of abscess formation may seem commonplace in many surgical procedures (such as an appendectomy in this case presentation), their potential to cause obstruction should not be overlooked [3,7].

Bowel obstruction secondary to a JP drain may arise due to direct mechanical compression, adhesion formation around the drain, or entrapment of bowel loops within the drain’s fenestrations. In our case, the patient developed progressive abdominal distention, bilious emesis, and persistently high output through her NG tube; all of which were initially suspected to be due to postoperative ileus, suggested by radiographs taken on postoperative day three. However, the patient’s failure to improve with conservative management prompted us to investigate this case further via an abdominal CT on postoperative day five, which ultimately identified a transition point in the right lower quadrant near the JP drain.

This case signifies the diagnostic challenges in distinguishing a true mechanical obstruction from other postoperative complications, including small bowel adhesion-induced obstruction or ileus. Imaging studies, such as CT scans, play a pivotal role in this scenario, not only identifying transition points but also guiding effective surgical management [11]. Furthermore, our patient’s delayed postoperative recovery despite conservative management reiterates the importance of holding a high index of suspicion for JP drain-related issues as part of one’s differential diagnosis when postoperative symptoms persist [7,8].

While the approximated incidence of JP drain-induced bowel obstruction is less than 1%, patient factors such as prior abdominal surgeries, inflammatory bowel disease, and malnutrition may increase the risk of this complication [12]. Despite prophylactic drainage remaining common practice post-GI surgeries, its routine use has been debated due to potential risks, one that is alleviated by judicious usage to not compromise any surgical outcomes [3,8]. This case also coincides with prior reports detailing JP drain-related complications where the drain acts as a “maypole” around which the small bowel loops twist, causing obstruction and, in severe cases, even necrosis [2,9]. 

Findings such as these underscore the importance of vigilance in drain placement and postoperative monitoring while simultaneously ensuring that prompt surgical intervention is undertaken to reduce mortality and ensure favorable outcomes. While some reports suggest that careful drain placement can prevent the risk of obstruction, there is a lack of consensus on specific surgical techniques to mitigate this complication [12]. Intraoperative strategies such as limiting drain placement near small loops of bowel, securing the drain away from mobile bowel segments, and early removal as clinically warranted can reduce the risk of entrapment. Moreover, selective drain use in cases with a low risk of postoperative fluid collection and abdominal cavity contamination should be considered to minimize potential complications. Further work into alternative drain strategies may help reduce the incidence of this rare yet devastating complication.

## Conclusions

Intraoperative strategies such as limiting drain placement near small loops of bowel, securing the drain away from mobile bowel segments, and early removal as clinically warranted can reduce the risk of entrapment. Selective drain use in cases with a low risk of postop fluid collection and abdominal cavity contamination should be considered to minimize potential complications. Further work into alternative drain strategies may help reduce the incidence of this rare yet devastating complication.
